# Supplementary material for: Defining Success in Open Science [version 2; peer review:2 approved]
Source: MNI Open Res. 2018 Mar 20;2:2. doi: 10.12688/mniopenres.12780.2 (PMC7839829; doi:10.12688/mniopenres.12780.2)
Supplement: Supplementary file 2 [file MNIOR-02-02-s002.docx]

Supplementary material 2

# Leadership Forum Participant List

**Dr. Sarah Ali-Khan,** Research Associate, Faculty of Law, McGill University

**Dr. Lluis Ballell-Pages,** Director, External Opportunities, GlaxoSmithKline, Tres Cantos Open Lab for Diseases of the Developing World

**Dr. Patricia Brennan,** Director, National Library of Medicine, Interim Associate Director for Data Science, National Institutes of Health, US Department of Health and Human Services

**Dr. Katja Brose,** Science Program Officer, Chan Zuckerberg Science Initiative

**Ms. Rachel Bruce,** Head of Open Science, Department for Business, Energy & Industrial Strategy, UK Government

**Mr. David Carr,** Program Manager - Open Research, Wellcome Trust

**Dr. Simon Chaplin,** Director of Culture and Society, Wellcome Trust

**Me Mylène Deschênes,** Ethics and Legal Advisor to Chief Scientist of Quebec, Fonds de recherche du Québec

**Dr. Aled Edwards,** Chief Executive Officer, Structural Genomics Consortium

**Ms. Ashley Farley,** Associate Officer of Knowledge & Research Services, Bill & Melinda Gates Foundation

**Dr. Richard Gold,** James McGill Professor, Faculty of Law, McGill University

**Ms. Jennifer Hansen,** Senior Officer, Knowledge & Research, Bill & Melinda Gates Foundation

**Dr. Jason Karamchandani,** Associate Professor, Department of Pathology, McGill University

**Dr. Michael Hawrylycz,** Investigator, Allen Institute for Brain Science

**Dr. Nadia Khelef,** Senior Advisor for Global Affairs, Institut Pasteur

**Mr. Robert J Kiley,** Head of Open Research Development, Wellcome Trust

**Ms. Elizabeth Kittrie,** Strategic Advisor for Data and Open Science, US National Institutes of Health

**Mr. Manoj Kumar,** Head of Entrepreneurship and Innovations, Tata Trusts

**Dr. Alexandre Le Bouthillier,** Founder and COO, Imagia

**Dr. Matthew Lucas,** Executive Director, Corporate Strategy and Performance, Social Sciences and Humanities Research Council of Canada

**Dr. Thomas Maina Kariuki,** Director of the Alliance for Accelerating Excellence in Science in Africa

**Dr. Lara Mangravite,** President, Sage Bionetworks

**Ms. Jessica Mankowski,** Manager, Knowledge Translation Strategies, Canadian Institutes of Health Research

**Dr. Sanjay Mehendale,** Additional Director General, Indian Council of Medical Research

**Dr. Mark Namchuk,** SVP Research, Non-Clinical and Pharmaceutical Development, Alkermes Biotech

**Ms. Thea Norman,** Senior Program Officer, Bill & Melinda Gates Foundation

**Mr. James O’Leary,** Chief Innovation Officer, Genetic Alliance

**Dr. Sébastien Paquet,** Lead Applied Research Scientist & Culture Hacker, Element AI

**Mr. Ben Pierson,** Senior Program Officer, Bill & Melinda Gates Foundation

**Dr. Claude Pirmez,** Senior Researcher, Oswaldo Cruz Institute

**Ms. Casey Selwyn,** Fellow, Global Health Program**,** Bill & Melinda Gates Foundation

**Ms. Annabel Seyller,** Chief Operating Officer of the Tanenbaum Open Science Institute (TOSI), McGill University

**Dr. Carthage Smith,** Senior Policy Analyst, Organization for Economic Co-Operation and Development (OECD)

**Dr. Jeff Spies,** Chief Technology Officer and Co-founder, Centre for Open Science

**Dr. David Sweeney,** Executive Chair Designate of Research England and Director, Research and Knowledge Exchange Higher Education Council for England

**Dr. Michiel van Den Hauten,** Head/Deputy Director of Research and Science Policy at the Ministry of Education, Culture and Science

**Dr. Kate Williams,** Scientific Director, Krembil Foundation
