# Supplementary material for: Defining Success in Open Science [version 2; peer review:2 approved]
Source: MNI Open Res. 2018 Mar 20;2:2. doi: 10.12688/mniopenres.12780.2 (PMC7839829; doi:10.12688/mniopenres.12780.2)
Supplement: Peer review history

# Current Peer Review Status:

**DOI:** 10.21956/mniopenres.13840.r26082

**Published:** 2018-03-07

**Authors:** Wen Hwa Lee

**Affiliations:** Structural Genomics Consortium (SGC), Nuffield Department of Medicine, University of Oxford, Oxford, UK

The authors have provided a very insightful and valuable report summarising the topics and outputs from the Open Science Leadership Forum in Washington, DC. It is evident from the report that the participants of the Forum are beyond the stage of simply advocating for Open Science (OS) and moving efficiently to structure OS as the norm, to make scientific endeavours more efficient and responsible.

The article covers many important advantages enabled by OS, including acceleration of innovation and impact, reproducibility, equity in research and positive economic impact. More importantly the authors have stated they would be developing an OS indicator toolbox and codebook to guide future implementations.

The authors also pointed out that this is the first of two reports – and perhaps in the second report the authors will map the perceived bottlenecks and propose mechanisms, strategies and policies to address barriers (for instance, exploring the incentives-rewards-recognitions in different dimensions involving science, i.e. academia, industry and society).

Finally, as OS can have different ‘flavours’ when implemented in different countries, fields and contexts it would be very useful to establish a mechanism whereby wider community can contribute either to the core discussion or to localised implementations. In this sense, I’d be more than happy and open to contribute and share in any of the discussions – and would encourage colleagues reading this article and this review to contribute generously to the debate.

**Is the rationale for the Open Letter provided in sufficient detail?**

Yes

**Does the article adequately reference differing views and opinions?**

Yes

**Are all factual statements correct, and are statements and arguments made adequately supported by citations?**

Yes

**Is the Open Letter written in accessible language?**

Yes

**Where applicable, are recommendations and next steps explained clearly for others to follow?**

Yes

***Competing Interests:*** No competing interests were disclosed.

**I have read this submission. I believe that I have an appropriate level of expertise to confirm that it is of an acceptable scientific standard.**

# Peer review

**DOI:** 10.21956/mniopenres.13840.r26078

**Published:** 2018-02-21

**Authors:** Gemma Hersh

**Affiliations:** Policy and Access team, Elsevier, London, UK

This article provides a thorough overview of the discussions that took place at the Leaders' Forum in Washington, DC to define success in Open Science. It provides a helpful summary of the indicators of Open Science discussed at that meeting and sets out a pathway for how these will be developed into a toolkit for measurement. There is a useful overview of how different actors perceive or anticipate OS to shape their particular world of research. Some early indications are provided of how measurement might be possible, with a recognition of likely difficulties in distinguishing between OS as a marker of success versus other variables.

While the report starts from the premise that the current system is unsustainable and unequal i.e. needs fixing, it would be helpful to briefly provide additional details or citations that point to a picture of the world of research today and its ideal state in the future.

**Is the rationale for the Open Letter provided in sufficient detail?**

Yes

**Does the article adequately reference differing views and opinions?**

Yes

**Are all factual statements correct, and are statements and arguments made adequately supported by citations?**

Partly

**Is the Open Letter written in accessible language?**

Yes

**Where applicable, are recommendations and next steps explained clearly for others to follow?**

Yes

***Competing Interests:*** No competing interests were disclosed.

**I have read this submission. I believe that I have an appropriate level of expertise to confirm that it is of an acceptable scientific standard.**
